# Oral microbiota and oral squamous cell carcinoma: a review of their relation and carcinogenic mechanisms

**DOI:** 10.3389/fonc.2024.1319777

**Published:** 2024-02-05

**Authors:** Bayu Indra Sukmana, Raed Obaid Saleh, Maryam Abdulrahman Najim, Hasan S. AL-Ghamdi, Harun Achmad, Mais Mazin Al-Hamdani, Abbas AY. Taher, Ali Alsalamy, Mansoor Khaledi, Kasra Javadi

**Affiliations:** ^1^ Oral Biology, Lambung Mangkurat, Banjarmasin, Indonesia; ^2^ Department of Medical Laboratory Techniques, Al-Maarif University College, Al-Anbar, Iraq; ^3^ Department of Biology, College Education for Pure Science, University of Anbar, Al-Anbar, Iraq; ^4^ Internal Medicine Department, Division of Dermatology, Faculty of Medicine, Albaha University, Albaha, Saudi Arabia; ^5^ Department of Pediatric Dentistry, Faculty of Dentistry, Hasanuddin University, Indonesia (Lecture of Pediatric Dentistry), Makassar, Indonesia; ^6^ Pharmaceutics Department, College of Pharmacy, Al-Ayen University, Thi-Qar, Iraq; ^7^ College of Dentistry, The Islamic University, Najaf, Iraq; ^8^ College of Technical Engineering, Imam Ja’afar Al‐Sadiq University, Al‐Muthanna, Iraq; ^9^ Department of Microbiology and Immunology, School of Medicine, Shahrekord University of Medical Sciences, Shahrekord, Iran; ^10^ Department of Microbiology, Faculty of Medicine, Shahed University, Tehran, Iran

**Keywords:** oral microbiota, oral squamous cell carcinoma, carcinogenic mechanisms, *Porphyromonas gingivalis*, *Fusobacterium nucleatum*, *Candida albicans*, human papillomavirus

## Abstract

Oral Squamous Cell Carcinoma (OSCC) is the most common type of head and neck cancer worldwide. Emerging research suggests a strong association between OSCC and the oral microbiota, a diverse community of bacteria, fungi, viruses, and archaea. Pathogenic bacteria, in particular *Porphyromonas gingivalis* and *Fusobacterium nucleatum*, have been closely linked to OSCC. Moreover, certain oral fungi, such as *Candida albicans*, and viruses, like the human papillomavirus, have also been implicated in OSCC. Despite these findings, the precise mechanisms through which the oral microbiota influences OSCC development remain unclear and necessitate further research. This paper provides a comprehensive overview of the oral microbiota and its relationship with OSCC and discusses potential carcinogenic pathways that the oral microbiota may activate or modulate are also discussed.

## Introduction

Oral squamous cell carcinoma (OSCC) ranks as the sixth most prevalent malignant tumor. Each year, approximately 263,000 new cases are detected worldwide. Tragically, the disease claims the lives of 127,000 individuals annually (1, 2). OSCC accounts for more than 90% of oral cancer (3, 4). The incidence of OSCC varies across different regions of the world, especially in relation to income levels and gender. The incidence has risen in low-income countries and among females. Furthermore, the incidence of OSCC in younger people especially those 45 years of age or younger has alarmingly increased (5, 6). The National Comprehensive Cancer Network (NCCN) states that OSCC may arise in the buccal mucosa, alveolar ridge, tongue, hard palate, retromolar trigone, floor of the mouth (FOM), and labial mucosa (7). The emergence of OSCC is linked to a number of risk factors. A few of the most prevalent risk factors include the use of tobacco products, drinking alcohol, eating betel nuts, nutritional inadequacies, poor oral hygiene, severe dental injuries, and HPV infection (2, 8, 9). The development of cancer in the pharynx and oral cavity may potentially be influenced by gene alterations. These genetic modifications have the potential to disrupt normal cellular activity by increasing the production of growth factors (such as transforming growth factor-α [TGF-α], TGF-β, platelet-derived growth factor, etc.) or the number of cell surface receptors (such as epidermal growth factor receptor, G-protein-coupled receptor, etc.). Additionally, they can enhance intracellular messenger signaling and lead to mutated production of transcription factors (such as the *ras* gene family, *c-myc* gene), which ultimately disrupts tightly regulated signaling pathways in normal cells. Oral cancer has been associated with the involvement of many oncogenes and tumor suppressor genes, including but not limited to the cyclin family, ras, PRAD-1, cyclin-dependent kinase inhibitors, p53, and RB1 (10). Recent studies have indicated that the oral microbiota, may also have an impact on the development and progression of OSCC (11, 12).

The oral cavity is home to a diverse and abundant microbiome, second only to the gut in terms of the number of microbial species present. This complex ecosystem is composed of over 700 different bacterial species, as well as fungi, viruses, and protozoa (13). A little over 54% of these have been cultivated and named, while 14% are cultivated but unnamed, according to the Human Oral Microbiome Database. The 32% that remain are just referred to as uncultivated phylotypes (14, 15). The oral cavity is a complex ecology with a variety of niches that enable various bacteria to preferentially colonize distinct habitats. Recent studies have shown the critical role the oral microbiota plays in preserving systemic and oral health. A healthy balance depends on the complex interactions that occur between the host and the many microbial populations that live in the mouth cavity (16).

Understanding the makeup, variety, and role of the oral microbiota is essential to developing methods that effectively prevent and cure cancer. An extensive examination of the oral microbiota and its connection to OSCC is provided by this review. It also describes possible carcinogenic pathways that might aid in the onset and advancement of OSCC.

## Oral bacteria associated with OSCC

Microorganisms were long ignored as a potential cause of cancer until interest in *Helicobacter pylori* as a causative agent of gastric cancer arose in the early 1990s. This discovery led to a paradigm shift in the medical community, with increased attention being paid to the role of infectious agents in cancer development (17, 18). Bacteria constitute the majority of the oral microbiota, while fungi and viruses account for a smaller proportion. Numerous oral bacterial species have been implicated in the development of oral cancer (19, 20). We will review oral bacteria’s function in OSCC in this study.

### Capnocytophaga gingivalis

One species of bacteria in the genus *Capnocytophaga* is *C*. *gingivalis*. The gram-negative, fusiform, fastidious, anaerobic, and microaerophilic bacteria belonging to the genus *Capnocytophaga* constitute a component of the oral flora of humans (21).

Unstimulated saliva was obtained from 229 OSCC-free and 45 OSCC individuals by Mager et al. For forty common oral bacteria, samples were analyzed. Three out of forty species, including *C*. *gingivalis*, were more prevalent in the saliva of OSCC patients (22). In another study, the salivary microbiome of 108 controls and 70 OSCC subjects was investigated (23). It was found that *C*. *gingivalis* was highly presented in OSCC tissues by fluorescence *in-situ* hybridization (FISH). This indicated that *C*. *gingivalis* might invade OSCC tissues.

The production of tissue-destroying hydrolytic enzymes by *C*. *gingivalis* causes the destruction of gingival soft tissue and alveolar bone. The aminopeptidase of *C*. *gingivalis* may be involved in the formation of bradykinin and the degradation of collagen fragments (24). LPS may either directly or indirectly promote the development and spread of cancer cells by inducing the host’s innate immune response (25). These biochemical results might all be connected to *C*. *gingivalis*’s tumor-promoting role in oral cancer.

Zhu et al. (23) found that *C*. *gingivalis* supernatant-stimulated OSCC cell lines showed fibroblastoid phenotypes and spindle-like shapes. Significant morphological alterations revealed decreased E-cadherin and β-catenin levels, whereas increased vimentin and SNAIL levels were observed. Additionally, these cells became more invasive and migratory. These findings suggest that *C*. *gingivalis* may accelerate OSCC by inducing tumor cell EMT.

### Fusobacterium

The genus *Fusobacterium* comprises several species of obligately anaerobic, non-spore-forming, motile or non-motile, Gram-negative rods (26). *Fusobacteria* are considered late colonizers of the healthy oral cavity (27). The emergence of OSCC has been associated with the *Fusobacterium nucleatum* species, which is commensal to the human oral cavity. The biofilms seen on OSCC are highly populated with anaerobic periodontal pathogens, such as *F*. *nucleatum*. This has led to the idea that these bacteria may play a part in the development of oral cancer (28, 29). Through a variety of processes, including the production of toxins, enzymes, and signaling molecules, this bacteria may cling to the oral epithelium, cause inflammation, alter the immune system, and encourage carcinogenesis (30). *F*. *nucleatum* connects its RadD adhesin to the streptococcal adhesin SpaP to connect with Streptococcus spp., which are early dental surface colonizers. This interaction also involves Aid1 and CmpA, two additional fusobacterial adhesins. *F*. *nucleatum*’s fusobacterial adhesins RadD, Fap2, and FomA bind to secondary colonizers such as *Porphyromonas gingivalis* on the biofilm. RadD also links *F*. *nucleatum* to *Actinomyces naeslundii* and *Candida albicans* (31).

Several investigations have shown that tissues impacted by OSCC contain *Fusobacterium*. By comparing cultured bacteria from OSCC tumor samples with healthy tissue samples from the same patients, Nagy et al. (1998) (32) discovered that OSCC tissue cultures had higher concentrations of *Fusobacterium* genera. In a different study, Zhao et al. (33) used next-generation sequencing to profile the bacteria in OSCC lesion surface samples at the species level in order to thoroughly examine the functional genes and makeup of the bacterial population in these samples. They discovered that there was a substantial enrichment of a set of taxa, including *Fusobacterium*, that were linked with periodontitis in OSCC samples. In 2017, Al-Hebsh et al. (34) sequenced DNA obtained from 20 fresh OSCC biopsies and 20 deep-epithelium swabs. They found that *F*. *nucleatum* subsp. *polymorphum* was the most significantly overrepresented species in the tumors. In order to confirm the link between periodontal infections and OSCC, researchers used fluorescence *in situ* hybridization, qPCR, and 16S rRNA amplicon sequencing in 2019. It was found that *F*. *nucleatum* was linked to subgingival plaques and was more common in cancerous tissue than in healthy tissue (35). Thirty healthy oral tissues were gathered along with tissues from fifty OSCC patients, and the frequency of *F*. *nucleatum* in both tumor and healthy tissue was assessed using a polymerase chain reaction. The findings demonstrated that, in contrast to healthy tissues, *F*. *nucleatum* was much more common in OSCC tissues (36).

*Fusobacterium* participates in OSCC formation and progression. *F*. *nucleatum* strongly induces collagenase 3, a matrix metalloproteinase that degrades extracellular matrix and aids tissue breakdown and cell migration. *F*. *nucleatum*-infected cells produce collagenase 3 through p38 MAPK, a signaling system that controls cell proliferation, survival, and inflammation. *F*. *nucleatum* may cause periodontal disorders and other infections by increasing collagenase 3 synthesis, epithelial cell migration, and survival (37).

*F*. *nucleatum* infection causes NF-κB translocation into the nucleus, resulting in cytokine gene production. Infection with F. nucleatum also turns on the NLRP3 inflammasome, which causes caspase-1 to be activated and mature IL-1β to be produced. Unlike other pathogens, *F*. *nucleatum* may be able to turn on the inflammasome in gingival epithelial cells (GECs) without ATP. At the same time that caspase-1 is activated, *F*. *nucleatum* infections release other damage-associated molecular patterns (DAMPs) that cause inflammation, such as high-mobility group box 1 protein and apoptosis-associated speck-like protein. *F*. *nucleatum* is known to make pathogen-associated molecular patterns that turn on NF-κB and act as an endogenous DAMP to activate the inflammasome. This causes more inflammation through secondary DAMPs (38).

The *F*. *nucleatum* infection of tongue squamous cell carcinoma cells (Tca8113) and its mechanisms were studied by Geng et al. The study found that *F*. *nucleatum* infection increased γH2AX expression, indicating DNA double-strand breaks, and boosted Tca8113 cell proliferation and cell cycle progression. The infection also decreased the expression of cyclin-dependent kinase inhibitor p27, DNA repair protein Ku70, and wild-type p53. Further results showed that Ku70 overexpression restored wild-type p53 and reduced *F*. *nucleatum*-infected Tca8113 cell growth. This suggests that *F*. *nucleatum* infection promotes OSCC by affecting DNA repair through the Ku70/p53 pathway and creating genomic instability (39).

Both oral epithelial cells and OSCC cells exhibit the process of epithelial-mesenchymal transition (EMT) when exposed to *F*. *nucleatum*. *F*. *nucleatum* infection increased migration of noncancerous human immortalized oral epithelial cells (HIOECs) and two OSCC cell lines (SCC-9 and HSC-4), but not proliferation or cell cycle. The infection affected EMT indicators such as E-cadherin, N-cadherin, vimentin, and snail family transcriptional repressor 1 (SNAI1) expression and location. *Fusobacterium nucleatum* adhesion A (FadA) adhesin and heat-inactivated version displayed comparable effects as live bacteria, suggesting that bacterial cell surface components were enough to induce EMT. Further work revealed that *F*. *nucleatum* infection elevated the long non-coding RNA (lncRNA) MIR4435-2HG, which negatively regulated miR-296-5p. In a dual-luciferase reporter experiment, MIR4435-2HG and miR-296-5p were shown to interact. The research also found that MIR4435-2HG knockdown decreased SNAI1 expression, and miR-296-5p inhibited Akt2, a downstream target and upstream regulator of SNAI1. Thus, *F*. *nucleatum* infection promoted EMT through the lncRNA MIR4435-2HG/miR-296-5p/Akt2/SNAI1 signaling pathway, indicating that oral epithelial carcinogenesis may be linked to EMT (40).

### Gemella

*Gemella* species are Gram-variable, facultatively anaerobic cocci that may form into pairs, clusters, or short chains (41).. These species are known to infrequently cause systemic illnesses and are a component of the oral microbiome in humans (42).

Researchers observed a strong correlation between the tumor location and two bacterial species, *Gemella haemolysans* and *Gemella morbillorum*, in a study that compared the oral microbiota in tumor and non-tumor tissues of individuals with OSCC (43). Further analysis using metagenomics revealed that the microbiome changed in association with the depth of invasion (DOI) of OSCC. Specifically, the abundances of several bacteria, including *Gemella haemolysans* and *Gemella morbillorum*, increased with increasing DOI, while the abundances of some other bacteria decreased (44). Certain studies imply that certain *Gemella* species could be involved in the emergence and spread of OSCC (43, 45).

### Lactobacillus

Lactobacilli are a type of commensal bacteria that are anaerobic, gram-positive, and rod-shaped. They are part of the normal flora found in the oral, genitourinary, and gastrointestinal tracts of the human body (46).

The variety and relative quantity of bacteria in the saliva of people with OSCC were investigated by Pushalkar et al. in their research. The study’s findings showed that *Lactobacillus* was one of the most common bacterial genera found in the saliva of OSCC participants (47). It is crucial to remember that the existence of these bacteria does not always indicate a causative association with OSCC, even if data implies that *Lactobacillus* is a common bacterial genus in people with OSCC. To completely comprehend the role these bacteria play in the development of OSCC, further study is necessary.

It has been discovered that bacteria of the genus Lactobacillus possess both carcinogenic and anticarcinogenic qualities. Lactic acid produced by these bacteria, along with other organic acids, acidifies the tumor microenvironment and aids in OSCC growth (48). It is known that changes in the activity of nicotinamide adenine dinucleotide phosphate (NADPH) oxidase and nitric oxide synthase (NOS) may cause the buildup of reactive oxygen species (ROS) and reactive nitrogen species (RNS), which in turn can cause chronic inflammation (49, 50). Some *Lactobacillus* species contribute to this process by generating hydrogen peroxide (H2O2) (51).

Conversely, research on human OSCC (HSC-3) has shown that geniposide, an anticancer drug, may be made to work even better by administering *Lactobacillus rhamnosus* GG (LGG) (52). Furthermore, it has been shown that *Lactobacillus plantarum* inhibits and activates the phosphatase and tensin homolog (PTEN) and mitogen-activated protein kinases (MAPKs) pathways, respectively, suggesting that it may regulate cancer. PTEN and MAPKs are known to be linked to the prevention and the start of cancer development, respectively. Consequently, There has been discussion on the potential use of *Lactobacillus plantarum* as a probiotic agent for the management of cancer (53, 54).

### Peptostreptococcus

Anaerobic bacteria of the *Peptostreptococcus* species are distinguished by their Gram-positive cocci form. It has also been discovered that these species are a typical component of the oral cavity’s flora (55).

In research by Lee et al. (56), there were notable differences found between individuals with epithelial precursor lesions and those without cancer in the oral microbiome compositions of five species, including *Peptostreptococcus*. The alterations in composition might potentially function as a biomarker to track the progression of oral carcinogenesis from a lesion of epithelium precursors to cancer. *Peptostreptococcus stomatis* was shown to be much more abundant in patients with OSCC in another investigation, indicating a possible link between the two conditions (12).

*P*. *stomatis* is capable of generating a variety of acids, including lactic, acetic, butyric, isobutyric, isovaleric, and isocaproic acids. These acids lower the pH of the surrounding environment. This reduction in pH creates a tissue microenvironment that is favorable for the proliferation and metastatic spread of cancer cells (57, 58).

### Porphyromonas

The genus *Porphyromonas* is composed of Gram-negative, asaccharolytic, obligately anaerobic, non-spore-forming, non-motile, and pleomorphic bacilli (59). One species within this genus is *P*. *gingivalis*, which belongs to the family *Porphyromonadaceae*. This bacterium is commonly found in the deep periodontal pockets of humans (60).

The study done by Nagy et al. and Pushalkar et al. (32, 47) revealed a notable disparity in the prevalence of the bacteria *Porphyromonas* between samples of OSCC and healthy samples. Chang et al. (35) looked into the connection between OSCC and periodontal infections using fluorescence *in situ* hybridization. The findings showed that malignant tissue had much greater *P*. *gingivalis* levels than normal tissue. Castañeda-Corzo et al. (61) performed research in which a population of 48 people was separated into two groups: 24 cases and 24 controls. A significant correlation was seen between the presence of *P*. *gingivalis* and the group of cases, with a detection rate of 66.7% among the patients.

The ability of *P*. *gingivalis* to survive and cause disease is heavily reliant on a wide range of virulence factors. These factors encompass both structural components inherent to the bacterium itself, such as lipopolysaccharide, fimbriae, and heat shock proteins, as well as secretory components like gingipains and outer membrane vesicles. The presence of these virulence factors allows *P*. *gingivalis* to effectively elude the host’s immune system and create a persistent infection, ultimately resulting in the development of periodontal disease (62).

*P*. *gingivalis* causes OSCC using different mechanisms. *P*. *gingivalis* excretes nucleoside-diphosphate-kinase (NDK), which promotes tumorigenesis. NDK inhibits ATP activation of purinergic receptor (P2X7) receptors, reducing IL-1β generation in epithelial cells. IL-1β is essential for priming IFNγ, which produces tumor-specific CD8+ T lymphocytes. Thus, *P*. *gingivalis* NDK may help tumors avoid immune surveillance. Apoptosis, which requires ATP activation of P2X7 receptors, is suppressed by NDK-mediated ATP degradation. Additionally, *P*. *gingivalis* NDK phosphorylates heat shock protein 27 (HSP27), which gives primary gingival epithelial cells an antiapoptotic phenotype (63, 64). Yilmaz et al. found that *P*. *gingivalis* inhibits epithelial cell death, which is both carcinogenic and protective for malignant cells. The Jak1/Akt/Stat3 signaling pathway, an increase in the Bcl2 (anti-apoptosis) to Bax (pro-apoptosis) ratio, a reduction in cytochrome c release, and the inhibition of caspase-9 and executioner caspase-3 may explain this progression. *P*. *gingivalis* also regulates microRNA expression. *P*. *gingivalis* upregulates miR-203, which lowers suppressor of cytokine signaling 3 (SOCS3), suppressing epithelial cell death. SOCS3 inhibits JAK/STAT3 signaling by binding to phosphorylated JAK receptors (65–67).

*P*. *gingivalis* causes persistent intracellular infections in epithelial cells. Due to the production of inflammatory molecules like IL-6, which cause DNA hypomethylation and promoter hypermethylation, chronic inflammation has been linked to cancer (68). Proinflammatory chemokine IL-8 may upregulate zinc-dependent matrix metalloproteinases, which degrade the extracellular matrix to help malignant epithelial cells metastasize. Through epidermal growth factor transactivation, IL-8 may also promote cell growth (69). Transforming growth factor β1 (TGF-β1) regulates several cellular functions. Numerous studies link TGF-β1 to epithelial-mesenchymal transition, tumor angiogenesis, and metastasis. TGF-β1, like IL-8, may boost OSCC cell invasiveness by activating matrix metalloproteinases (70). Tumor necrosis factor α (TNF-α), a cytokine, affects carcinogenesis at several stages. Reactive oxygen species (ROS) and reactive nitrogen intermediates (RNI) from this protein cause genomic instability and cancer-causing mutations. Other TNF-α pathways include inducing epithelial-mesenchymal transition and vascular endothelial growth factor (VEGF) production, which promotes tumor angiogenesis (71). *P*. *gingivalis* infection promotes B7-H1 receptor expression, which regulates cell-mediated immune responses. This implies *P*. *gingivalis* may transmit cancer cells to distant places and advance nuclear grading. However, B7-H1 receptor-mediated costimulatory signals may induce anergy and apoptosis in activated T cells, enabling malignancies to avoid immunological responses (72, 73).

*P*. *gingivalis* metabolites may cause cancer. Oxygen radicals, butyrate, and acetaldehyde are metabolites. Butyrate is a strong bacterial carcinogen that may cause T- and B-lymphocyte apoptosis and decrease their function. Mutations and genomic instability may result from oxygen radical-induced DNA damage such double-strand breaks or base alterations. A hazardous byproduct of ethanol metabolism, acetaldehyde may damage DNA and promote aberrant epithelial cell growth. *P*. *gingivalis* may cause OSCC by creating these toxic metabolites (74, 75).

### Prevotella

*Prevotella*, a genus of Gram-negative bacteria, plays a significant role in the microbial ecosystem of the oral cavity (76). These non-motile, single-celled organisms flourish under anaerobic growth conditions (77). Two species within this genus, *Prevotella intermedia* and *Prevotella melaninogenica*, have been identified as critical players in oral health. Recent studies have revealed a potential association between these species and OSCC, highlighting their importance in oral pathology (45, 78).

Hsiao et al. (79) used targeted 16S rRNA gene sequencing to evaluate oral bacterial profiles. *P*. *intermedia* was detected in 2.16% of OSCC patients. The research also indicated that *P*. *intermedia* enhanced OSCC risk. Biopsies from OSCC tumors were taken in another case-control investigation. The research included 24 patients (cases) and 24 controls, totaling 48 donors over 55. P. *intermedia* was discovered in 83.3% of patients using q-PCR. These data imply that *P*. *intermedia* may cause OSCC (61). Mager et al. (22) found higher counts of three bacteria, including *P*. *melaninogenica*, in OSCC patients’ saliva. These three species have 80% sensitivity in predicting cancer cases and 83% specificity in eliminating non-matched controls as diagnostic indicators. These data imply that elevated salivary *P*. *melaninogenica* numbers may indicate OSCC.

Pathogen *P*. *intermedia* is recognized for producing virulence factors, including lipoteichoic acids, peptidoglycans, fimbriae adhesins, and LPSs. At different phases of OSCC, these variables have been shown to cause chronic inflammation of the oral cavity by inducing the production of pro-inflammatory cytokines (12, 80).

Repeated periodontitis, caused by *P*. *intermedia*, has been linked to OSCC. This bacteria secretes peptides, including proteases that activate proteinase-activated receptors to signal. This activation affects cell proliferation, apoptosis, autoimmunity, cytokine generation, microenvironment inflammation, pain, and epithelial barrier function. Cancer areas had more peptidases, according to functional analyses. Tumors may develop and grow when bacterial proteases damage host tissue like the extracellular matrix (ECM), alter host physical barriers, and affect the host immunological response (12).

*P*. *intermedia* produces halitosis-causing volatile sulfur compounds, mostly hydrogen sulfide and methyl mercaptan. Both oxidative stress and DNA damage in oral cells have been linked to these substances. Hydrogen sulfide may block superoxide dismutase, which prevents ROS accumulation in human cells, even at low doses. Methyl mercaptan breaks down type 4 collagen and may help OSCC invade the basement membrane (81).

### Streptococcus

*Streptococcus* is a genus of Gram-positive bacteria belonging to the family *Streptococcaceae* (82). One species of *Streptococcus*, *Streptococcus anginosus*, is a member of the oral viridans streptococci and is commonly found in dental plaque as a commensal bacterium (83). *Streptococcus gordonii*, a Gram-positive bacteria, is known to exist as a commensal organism in several regions of the human body, such as the skin, oral cavity, and gut (84). Additionally, *Streptococcus mitis* is one of the earliest commensal colonizers of the human oral cavity (85). These bacteria may affect human health in both positive and negative ways, and they are crucial for preserving the equilibrium of the human microbiome.

Studies by Nagy et al. and Pushalkar et al. have shown that samples from OSCC patients had a much higher prevalence of the bacteria *Streptococcus* than do those from healthy persons (32, 47). Sasaki et al. (86) found *S*. *anginosus* DNA in 19 of 42 squamous cell cancer samples. It was absent from lymphoma, rhabdomyosarcoma, and leukoplakia samples. All 19 squamous cell carcinoma cases that tested positive for *S*. *anginosus* had the bacteria solely in dental plaque, not saliva. Additionally, the genotype of *S*. *anginosus* in cancer tissue matched that in dental plaque from the same individuals. *S*. *anginosus* infection may be frequent in OSCC, and tooth plaque may be a reservoir for the bacteria. Torralba et al. (87) used PCR to identify *S*. *gordonii* and other oral bacteria in OSCC tumor tissue (TT), non-tumor tissue (NT), and saliva (SA) samples from 18 OSCC patients. *S*. *gordonii* was found in 72.2% of TT, 33.3% of NT, and 38.9% of SA samples, indicating a link between this bacterium and OSCC. Researchers examined the salivary counts of 40 common oral bacteria in people with OSCC to those in cancer-free controls in an effort to investigate any possible connections between oral bacteria and OSCC. The results showed that the saliva of people with OSCC had a significantly higher concentration of *S*. *mitis*. As a result, the research came to the conclusion that high *S*. *mitis* salivary numbers may be useful as diagnostic markers for OSCC (22).

*S*. *anginosus* has been found to contribute to the carcinogenesis of OSCC through several mechanisms. One such mechanism is through the induction of DNA damage in the oral mucosa. This damage is caused by increased synthesis of nitric oxide (NO) and cyclooxygenase 2 (COX-2), both of which are produced by S. anginosus (80, 88). COX-2 is an enzyme that catalyzes the rate-limiting step in the synthesis of prostaglandins, which are involved in inflammation and pain. Overexpression of COX-2 has been associated with cancer metastasis and poor clinical outcomes (89).

*S*. *gordonii* activates a signaling cascade including transforming growth factor-β-activated kinase 1 (TAK1) and nemo-like kinase 1 (Nlk1) to phosphorylate forkhead box protein O1 (FOXO1). The enzymes add phosphate groups to FOXO1, causing it to depart the nucleus and lose function. Thus, FOXO1 cannot influence zinc finger E-box–binding homeobox (ZEB2) gene expression. ZEB2, a transcription factor, induces EMT. EMT makes cells more mobile and invasive by changing their shape and behavior (90). *S*. *gordonii* plays a role in metabolizing alcohol into acetaldehyde, a substance with carcinogenic potential. Due to their ability to produce acetaldehyde, these bacteria may pose a risk for the development of OSCC (54).

*S*. *mitis* has been shown to inhibit the proliferation of OSCC tumor cells through its cytotoxicity, which is mediated by the production of hydrogen peroxide (91). It is crucial to remember that *S*. *mitis* is a large generator of acetaldehyde, a recognized carcinogen, even though it may have a preventive effect against OSCC (92).

### Treponema denticola

*T*. *denticola* is a Gram-negative, motile, and obligate anaerobic bacterium that is commonly associated with periodontal lesions (93). This highly proteolytic spirochete lives in the oral cavity of humans and has been found to be predominant in periodontal lesions of adult periodontitis (94).

In a study by Kaliamoorthy et al. (95) a total of 30 OSCC and oral mucosal non-cancerous tissue specimens were collected from patients and controls, respectively. The results showed that *T*. *denticola* was detected in 8 OSCC tissue samples, while no control tissue specimen was found to be positive for the bacterium. These results imply that *T*. *denticola* may be involved in the pathogenesis of OSCC.

*T*. *denticola* major secreted protease, dentilisin, plays a vital role in tumor invasion and development. Dentilisin may affect the inflammatory and matrix remodeling responses by degrading cytokines (IL-8 and TNFα) and activating matrix metalloproteinases (MMP8 and MMP9). Moreover, dentilisin may overcome the inhibitory effects of tissue inhibitors of MMPs (TIMP1 and TIMP2), generating a more conducive environment for epithelial invasion. Dentilisin expression is substantially connected with early-stage mobile tongue squamous cell carcinoma, and is associated with poorer prognosis in individuals younger than 60 years of age (69, 96).

### Other bacteria

**Figure 1:**
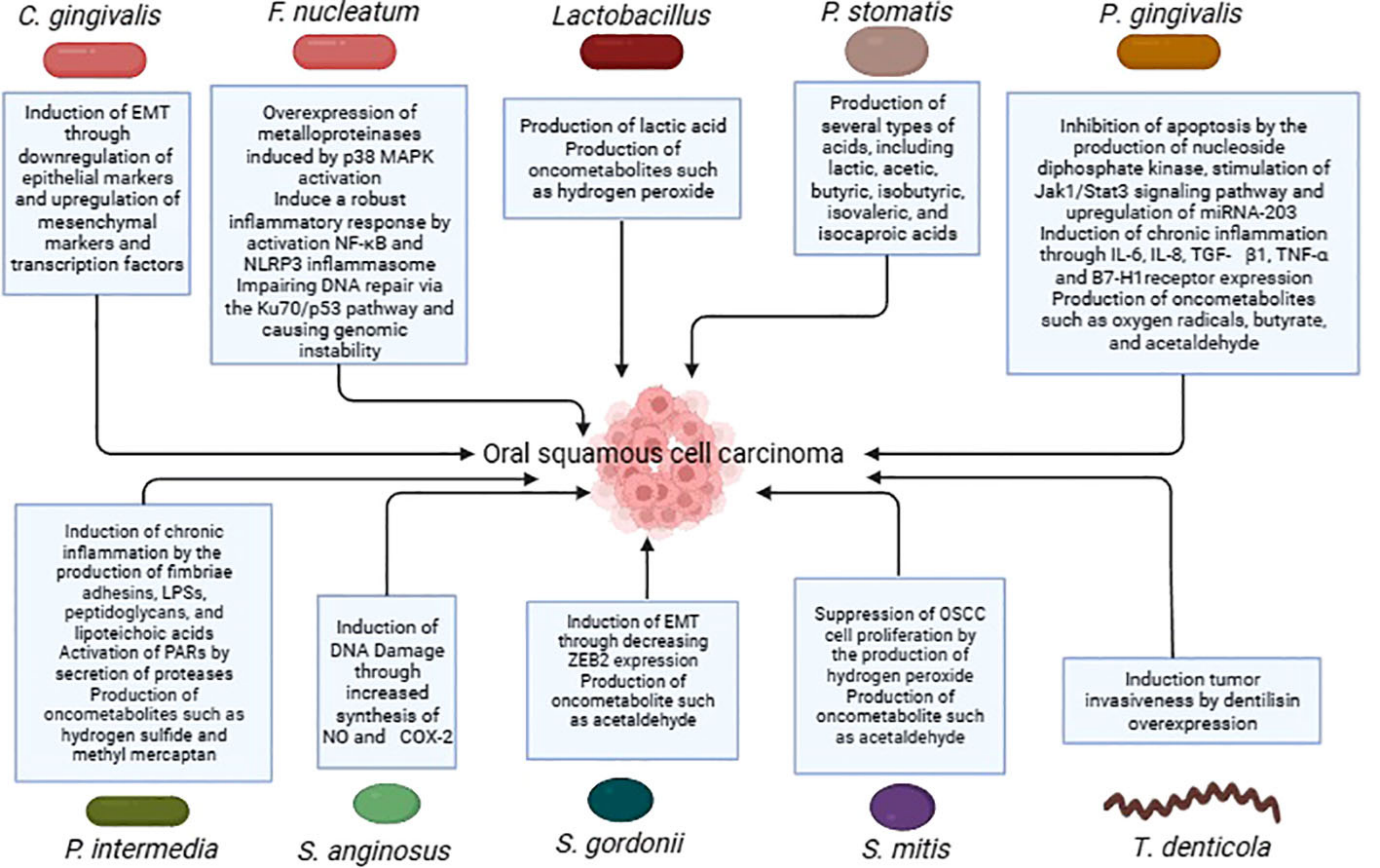
Carcinogenic mechanisms of oral bacteria in OSCC.

Several more oral bacteria have also been shown to be carcinogenic, in addition to the previously listed ones. In comparison to healthy persons, oral cancer samples showed a higher variety of bacterial species, as per a research conducted by Zhao et al. In particular, it was discovered that OSCC samples from people who had previously had periodontitis had overexpressed levels of the bacteria *Catonella*, *Dialister*, *Filifactor*, and *Parvimonas* (33). Significant variations in the microbial makeup and profile were seen between matched paracancerous tissues and OSCC lesions in a different research conducted by Zhou et al. Bacteria belonging to the genera *Carnobacterium*, *Tannerella*, *Parvimonas*, and *Filifactor* were linked to malignant tumors (97). **Table 1** summarises the studies conducted on oral bacteria in OSCC patients, while the carcinogenic mechanisms of oral bacteria are elucidated in **Table 2** and **Figure 1**.

**Table 1:** Oral bacteria in OSCC patients.

Genus	Species	Specimen type	Detection method	Reference
*Capnocytophaga*	*gingivalis*	Saliva	Checkerboard DNA-DNA hybridization	Mager et al., 2005 (22)
		Tissue	16S rRNA sequencing	Zho et al., 2022 (23)
*Fusobacterium*	*-*	Tissue	Cell culturing	Nagy et al., 1998 (32)
		Tissue	Metagenomic sequencing	Zhao et al., 2017 (33)
	*Nucleatum*	Tissue	16S rRNA sequencing	Al-Hebshi et al., 2017 (34)
		Tissue	16S rRNA sequencing	Chang, et al., 2019 (35)
		Tissue	PCR	Mandal et al., 2021 (36)
*Gemella*	*haemolysans/morbillorum*	Tissue	16S rRNA sequencing	Pushalkar et al., 2012 (43)
		Tissue	Metagenomic sequencing	Liu et al., 2022 (44)
*Lactobacillus*	–	Saliva	16S rRNA pyrosequencing	Pushalkar et al., 2011 (47)
*Peptostreptococcus*	–	Saliva	16S rRNA sequencing	Lee et al., 2017 (56)
	*Stomatis*	Tissue	16S rRNA sequencing	Zhang et al., 2020 (12)
*Porphyromonas*	*-*	Tissue	Cell culturing	Nagy et al., 1998 (32)
		Saliva	16S rRNA pyrosequencing	Pushalkar et al., 2011 (47)
	*Gingivalis*	Tissue	16S rRNA sequencing	Chang et al., 2019 (35)
		Tissue	qPCR	Castañeda-Corzo et al., 2023 (61)
*Prevotella*	*Intermedia*	Saliva	16S rRNA sequencing	Hsiao et al., 2018 (79)
		Tissue	qPCR	Castañeda-Corzo et al., 2023 (61)
	*melaninogenica*	Saliva	Checkerboard DNA-DNA hybridization	Mager et al., 2005 (22)
*Streptococcus*	*-*	Tissue	Cell culturing	Nagy et al., 1998 (32)
		Saliva	16S rRNA pyrosequencing	Pushalkar et al., 2011 (47)
	*Anginosus*	Tissue	PCR	Sasaki et al., 2005 (86)
	*Gordonii*	Tissue	PCR	Torralba et al., 2021 (87)
	*mitis*	Saliva	Checkerboard DNA-DNA hybridization	Mager et al., 2005 (22)
*Treponema*	*Denticola*	Tissue	PCR	Kaliamoorthy et al., 2021 (95)

**Table 2:** Carcinogenic mechanisms of oral bacteria.

Oral bacteria	Carcinogenic mechanisms	Reference
*C*. *gingivalis*	Induction of EMT through downregulation of epithelial markers and upregulation of mesenchymal markers and transcription factors	(23)
*F. nucleatum*	Overexpression of metalloproteinases induced by p38 MAPK activation	(98)
	Induce a robust inflammatory response by activation NF-κB and NLRP3 inflammasome	(38)
	Impairing DNA repair via the Ku70/p53 pathway and causing genomic instability	(39)
	Induction of EMT via the lncRNA MIR4435-2HG/miR-296-5p/Akt2/SNAI1 signaling pathway	(40)
*Lactobacillus*	Production of lactic acid	(48)
	Production of oncometabolites such as hydrogen peroxide	(51)
*P. stomatis*	Production of several types of acids, including lactic, acetic, butyric, isobutyric, isovaleric, and isocaproic acids	(57, 58)
*P*. *gingivalis*	Inhibition of apoptosis by the production of nucleoside diphosphate kinase, stimulation of Jak1/Stat3 signaling pathway and upregulation of miRNA-203	(63–67)
	Induction of chronic inflammation through IL-6, IL-8, TGF-β1, TNF-α and B7- H1receptor expression	(68–71, 73)
	Production of oncometabolites such as oxygen radicals, butyrate, and acetaldehyde	(74, 75)
*P. intermedia*	Induction of chronic inflammation by the production of fimbriae adhesins, LPSs, peptidoglycans, and lipoteichoic acids	(12, 80)
	Activation of PARs by secretion of proteases	(12)
	Production of oncometabolites such as hydrogen sulfide and methyl mercaptan	(81)
*S*. *anginosus*	Induction of DNA Damage through increased synthesis of NO and COX-2	(80)
*S. gordonii*	Induction of EMT through decreasing ZEB2 expression	(90)
	Production of oncometabolite such as acetaldehyde	(54)
*S*. *mitis*	Suppression of OSCC cell proliferation by the production of hydrogen peroxide	(91)
	Production of oncometabolite such as acetaldehyde	(92)
*T*. *denticola*	Induction tumor invasiveness by dentilisin overexpression	(69)

### Fungi associated with OSCC

While the bacterial elements of the OSCC microbiome have been extensively studied, the fungal components remain relatively unexplored and poorly defined (99). The mycobiome is an essential part of the human microbiome; it is the collective genome of different fungus species. Even though they make up a very minor portion of the oral microbiota, fungus may affect host health in a variety of ways (100). In the following, we will delve into the role of fungi in OSCC.

### Candida

*Candida* is a genus of yeast-like fungi that is frequently studied in relation to OSCC. These fungi can cause oral candidiasis, commonly known as thrush. While *C*. *albicans* is the most prevalent species, other species have also been found in OSCC patients (101).

Researchers found that 72.2% of patients with OSCC had Candida species in their saliva. Interestingly, non-*C*. *albicans* was found to be more prevalent than *C*. *albicans* (102). Another study showed an increase in the genera *Candida* among OSCC lesions, with *C*. *albicans* and *Candida etchellsii* being the most enriched species in OSCC (103).

The potential carcinogenic mechanisms of *C*. *albicans* are multifaceted. The first step in the colonization and invasion of *C*. *albicans* is adhesion to mucosal epithelial cells, which serve as the initial line of protection against microbes. This adhesion can weaken the host’s defense mechanisms, allowing for further infection (104). Additionally, it has been shown that *C*. *albicans* produces carcinogens such nitrosamines, which have the ability to activate proto-oncogenes and cause carcinomatous alterations (105). A *C*. *albicans* infection-related chronic inflammation may also play a role in the development of cancer. Moreover, *C*. *albicans* may cause carcinogenesis by triggering the Th17 response. Th17 cells are a subset of helper T cells that are important for maintaining mucosal barriers and aiding in the removal of pathogens from mucosal surfaces (106).

### Other fungi

**Figure 2:**
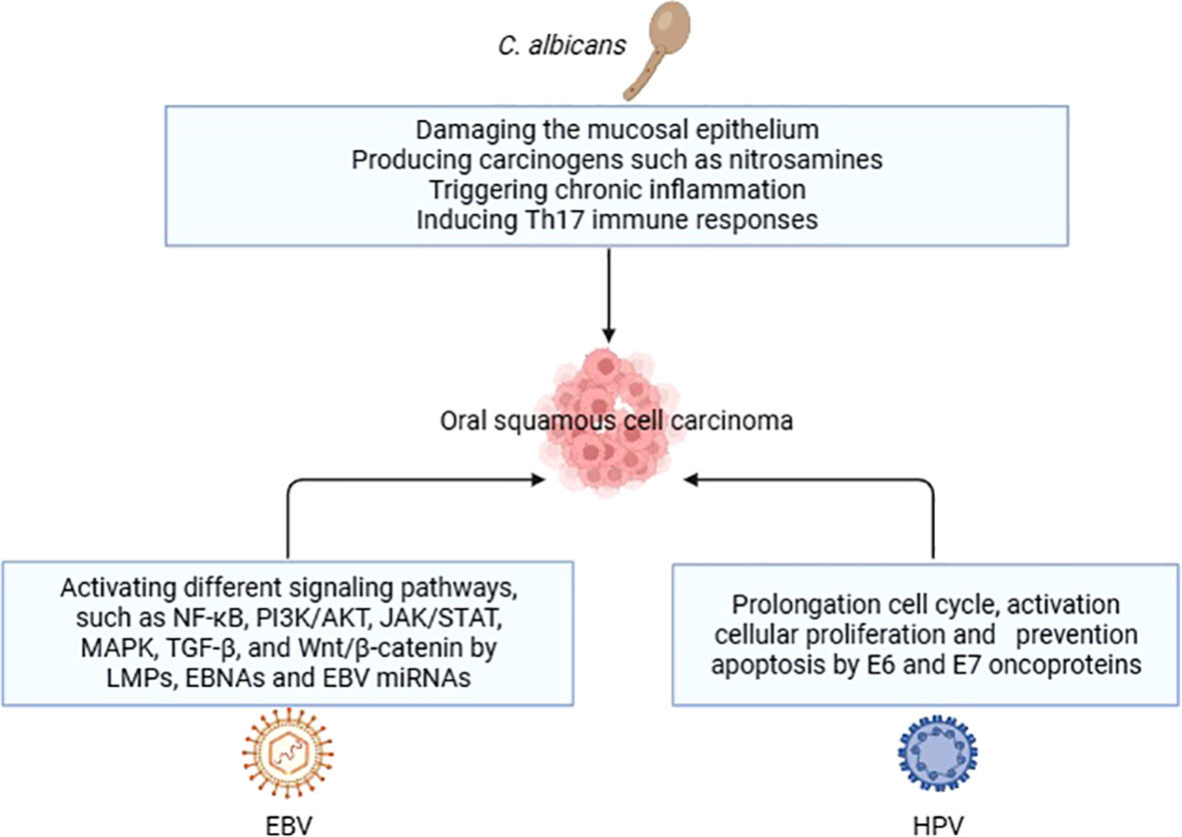
Carcinogenic mechanisms of oral fungi and viruses in OSCC.

According to a research done by Perera et al. (103) fungus belonging to the genera *Hannaella* and *Gibberella* were found to be considerably more numerous in samples of OSCC when compared to fibroepithelial polyp (FEP) controls. However, another study failed to identify the presence of *Hannaella* and *Gibberella* in a cohort of OSCC patients, leading researchers to consider them as contaminants. These conflicting results may indicate the influence of factors such as dietary habits and population-related differences in the mycobiome profiles of individuals (101). **Table 3** summarizes the studies conducted on oral fungi in OSCC patients, while **Table 4** and **Figure 2** illustrate the carcinogenic mechanisms of oral fungi.

**Table 3:** Fungi in OSCC patients.

Genus	Species	Specimen type	Detection method	Reference
*Candida*	*spp*.	Saliva	PCR–RFLP	Sankari et al., 2020 (102)
		Tissue	DNA sequencing	Perera et al., 2017 (103)
*Gibberella*/ *Hannaella*	*spp*.	Tissue	DNA sequencing	Perera et al., 2017 (103)

**Table 4:** Carcinogenic mechanisms of fungi.

Fungi	Carcinogenic mechanisms	Reference
*C*. *albicans*	Damaging the mucosal epithelium	(104)
	Producing carcinogens such as nitrosamines	(105)
	Triggering chronic inflammation	(106)
	Inducing Th17 immune responses	(106)

## Viruses associated with OSCC

Viruses, which are a component of the oral microbiota, have been related to several disorders affecting the oral cavity (107). In the early 20th century, Peyton Rous discovered that viruses can cause cancer (108). Research has shown that viruses may play a role in the development of OSCC. While some viruses have a strong association with OSCC, others have a less frequent association and may require cofactors to exert their carcinogenic effects. As such, it is important to carefully evaluate the role of viruses in OSCC in order to improve diagnosis and treatment (109).

Viral infections have the potential to enhance genetic diversity and phenotypic flexibility, hence expediting the primary mechanisms underlying the genesis of cancer cells. This phenomenon is often known as biological entropy. Viruses have the ability to impact the criteria for evolutionary selection by modifying both the tumor microenvironment and immunological signals. Furthermore, the presence of tumor heterogeneity has been identified in malignancies linked to different viruses, as shown by variations in viral and host gene expression. This heterogeneity has been explored using techniques such as single-cell RNA sequencing, analysis of epigenetic alterations, and examination of immune infiltration. This finding suggests that the presence of heterogeneity is a prevailing characteristic in both viral and non-viral forms of cancer (110).

### Epstein-Barr virus (EBV)

Human oncogenic virus EBV has been connected to the development of cancer in epithelial and lymphoid cells (111). However, it’s important to note that the relationship between past EBV infection and OSCC is not entirely clear. One study found that 96.6% of OSCC patients and 97.2% of control subjects tested positive for EBV VCA IgG, an indicator of past EBV infection. This suggests that past EBV infection may not play a significant role as a risk indicator for OSCC (112).

According to a research by Francesco Broccolo et al. (113) a greater incidence of EBV was discovered in OSCC patients, with 72.7% of the patients testing positive for the virus. The mean EBV copy number was higher in OSCC tissue samples than in oral lichen planus (OLP) and oral irritation fibroma (OIF) samples in another study that examined EBV, viral load, and EBV-encoded small RNA (EBER) sequence variation in tissue samples from patients with OSCC and other oral cavity lesions. Difference was not statistically significant. The research found more than one genome copy per tumor cell, suggesting EBV infection in oral malignancies (111).

EBV may cause oncogenesis by triggering several signaling pathways in the host cell. Among these pathways are transforming growth factor-β (TGF-β), Janus kinase/signal transducer and transcription activator (JAK/STAT), phosphoinositide-3-kinase/protein kinase B (PI3K/AKT), nuclear factor-κB (NF-κB), and Wnt/β-catenin. EBV-encoded proteins and noncoding RNA, including as latent membrane proteins (LMPs), Epstein-Barr nuclear antigens (EBNAs), and EBV miRNAs, regulate these processes. Due to their ability to alter cellular processes and stimulate the formation of tumors, these chemicals are essential in the development of cancer (114, 115).

### Human papilloma virus (HPV)

HPV has the ability to infect several mucosal and dermal regions of the human body, resulting in the formation of both benign and malignant lesions. Although HPV is commonly acknowledged as the predominant etiological factor for cervical cancer, its involvement in the pathogenesis of other malignancies, such as OSCC, has also been reported (116, 117).

According to a research by Zhao et al. (118) 40.4% of the tumors tested positive for HPV, with HPV16 making up 63.5%, HPV18 accounting for 30.8%, HPV6 accounting for 3.9%, and HPV11 accounting for 1.8%. This shows that HPV infection may function as a stand-alone predictor of OSCC survival and prognosis. In a different investigation, no blood samples tested positive for high-risk HPV, however two saliva samples (15.4%) and one tissue sample (1.6%) tested positive for HPV16 and HPV18, respectively. These results suggest that, as OSCC linked with high-risk HPV has a better prognosis, identifying high-risk HPV in patients may help with patient management decisions (119).

Two oncoproteins, E6 and E7, which are encoded by HPV, are directly involved in the progression of HPV-induced carcinogenesis. Together, these oncoproteins target several cellular pathways that govern apoptosis, cell polarity control networks, and cell cycle regulation (120). As a result of the actions of E6 and E7, infected cells are prevented from undergoing apoptosis and remain in a state of persistent cell cycle activity. This ability can be attributed to the interaction between the proteins RB1, RBL1, and E7, as well as the degradation of TP53 induced by E6 (20).

### Other viruses

Furthermore, it has been postulated that Human Cytomegalovirus (HCMV) and Herpes Simplex Virus-1 (HSV-1), in addition to the viruses previously discussed, are associated with the pathogenesis of OSCC. According to a research conducted by Saravani et al. (121) it was postulated that HCMV may have a substantial impact on the development of OSCC, given its frequent presence in the gingival sulcus fluid. Another study discovered that 24 out of 155 OSCCs examined (15%) tested positive for HSV-1 (122). **Table 5** compiles the studies conducted on oral viruses in OSCC patients, while the carcinogenic mechanisms of oral viruses are depicted in **Table 6** and **Figure 2**.

**Table 5:** Viruses in OSCC patients.

Virus Type	Specimen type	Detection method	Reference
EBV	Tissue	qPCR	Broccolo et al., 2018 (113)
	Tissue	qPCR	Zebardast et al., 2021 (111)
HPV	Tissue	nested PCR	Zhao et al., 2009 (118)
	Saliva/Blood/Tissue	nested PCR	Fauzi et al., 2019 (119)

**Table 6:** Carcinogenic mechanisms of viruses.

Viruses	Carcinogenic mechanisms	Reference
EBV	Activating different signaling pathways, such as NF-κB, PI3K/AKT, JAK/STAT, MAPK, TGF-β, and Wnt/β-catenin by LMPs, EBNAs and EBV miRNAs	(114, 115)
HPV	Prolongation cell cycle, activation cellular proliferation and prevention apoptosis by E6 and E7 oncoproteins	(120)

## Conclusions

The oral microbiota is comprised of a diverse and ever-changing assemblage of bacteria that reside inside the mouth cavity and engage in interactions with both the host and the surrounding environment. The oral microbiota has the potential to have influence on both oral health and illness, as well as systemic disorders, including cancer. OSCC is the prevailing form of oral malignancy. This study offers a thorough examination of the associations among oral bacteria, fungi, and viruses in relation to OSCC. Furthermore, this paper provides an overview of the various processes via which certain oral bacteria may contribute to the development of oral carcinogenesis. Nevertheless, there is a lack of comprehensive understanding of the precise involvement of oral microbiota in the onset and progression of OSCC. The objective of this study is to examine the significance of the oral microbiota in patients with OSCC, along with the corresponding processes of carcinogenesis. Understanding these factors might potentially aid in the identification of disease progression or recurrence, and perhaps enhance treatment results.

In the future, researchers may try to learn more about how certain bacteria in the mouth cause OSCC. Additionally, there is a need to explore new techniques for prevention or treatment that specifically target these bacteria. Also, creating diagnostic models based on the link between OSCC and the oral microbiota could lead to less invasive and more cost-effective ways to find cancer.

## Author contributions

BS: Conceptualization, Project administration, Resources, Software, Supervision, Validation, Writing – original draft, Writing – review & editing. RS: Investigation, Methodology, Software, Validation, Writing – original draft, Writing – review & editing. MN: Investigation, Methodology, Validation, Writing – original draft, Writing – review & editing. HA-G: Investigation, Methodology, Validation, Writing – original draft, Writing – review & editing. HA: Investigation, Methodology, Validation, Writing – original draft. MA-H: Investigation, Resources, Validation, Writing – original draft. AT: Investigation, Resources, Validation, Writing – original draft. AA: Data curation, Investigation, Validation, Writing – original draft. MK: Conceptualization, Investigation, Methodology, Project administration, Resources, Supervision, Validation, Visualization, Writing – original draft, Writing – review & editing. KJ: Investigation, Methodology, Project administration, Resources, Software, Supervision, Validation, Visualization, Writing – original draft, Writing – review & editing.
